# Vegetable Exudates as Food for *Callithrix* spp. (Callitrichidae): Exploratory Patterns

**DOI:** 10.1371/journal.pone.0112321

**Published:** 2014-11-05

**Authors:** Talitha Mayumi Francisco, Dayvid Rodrigues Couto, José Cola Zanuncio, José Eduardo Serrão, Ita de Oliveira Silva, Vanner Boere

**Affiliations:** 1 Departamento de Biologia Animal, Universidade Federal de Viçosa, 36570-900, Viçosa, MG, Brazil; 2 Departamento de Botânica/Museu Nacional, Universidade Federal do Rio de Janeiro, 20940-040, Rio de Janeiro, RJ, Brazil; 3 Departamento de Biologia Geral, Universidade Federal de Viçosa, 36570-900, Viçosa, Brazil; 4 Departamento de Bioquímica e Biologia Molecular, Universidade Federal de Viçosa, 36570-900, Viçosa, Brazil; University of Florence, Italy

## Abstract

Marmosets of the genus *Callithrix* are specialized in the consumption of tree exudates to obtain essential nutritional resource by boring holes into bark with teeth. However, marmoset preferences for particular tree species, location, type, and other suitable factors that aid in exudate acquisition need further research. In the current study, the intensity of exudate use from *Anadenanthera peregrina* var. *peregrina* trees by hybrid marmosets *Callithrix* spp. groups was studied in five forest fragments in Viçosa, in the state of Minas, Brazil. Thirty-nine *A. peregrina* var. *peregrina* trees were examined and 8,765 active and non-active holes were analyzed. The trunk of *A. peregrina* var. *peregrina* had a lower number of holes than the canopy: 11% were found on the trunk and 89% were found on the canopy. The upper canopy was the preferred area by *Callithrix* spp. for obtaining exudates. The intensity of tree exploitation by marmosets showed a moderate-to-weak correlation with diameter at breast height (DBH) and total tree height. The overall results indicate that *Anadenanthera peregrina* var. *peregrina* provides food resources for hybrid marmosets (*Callithrix* spp.) and these animals prefer to explore this resource on the apical parts of the plant, where the thickness, location, and age of the branches are the main features involved in the acquisition of exudates.

## Introduction

Vegetable exudates, such as saps and gums, are constitutive or induced compounds, which are essential components of the diet of many primates [1]. To date, at least 69 species of primates, representing 12 families, are known to consume plant exudates [2], being either ‘nonspecialists’ or ‘specialists’ feeders [3]. Specialist species include marmosets *Callithrix* spp. Erxleben, 1777 (Cebidae); *Mico* Lesson, 1840 (Cebidae); *Cebuella pygmaea* Spix, 1823 (Cebidae); *Phaner* Gray, 1870 (Cheirogaleidae); *Euoticus* Gray, 1863 (Galagidae) and slow lorises *Nycticebus* spp. É. Geoffroy, 1812 (Lorisidae) [2], [4], [5]. These genera are obligatory consumers of plant exudates [1], [3]; they have evolved anatomical adaptations of their teeth and of the bones and muscles in their skull to perforate plant branches to stimulate the release of exudates [6], [7]. This behavior has been termed ‘gouging.’

Within the Primates order, members of the New World subfamily Callitrichinae perhaps consume the largest amount of exudates [3], [4], [8]. In particular, the genera *Callithrix*, *Cebuella*, and *Mico* are specialized in the acquisition of this nutritional resource. During specific seasons, exudates can comprise up to 70% of the diet of *Callithrix* spp. [9], [10], with 30% of the daily activity of these species spent gouging [3], [11]. *Callithrix* species have evolved functional characteristics that enable them to extract exudates from plants [12], [14], including a specialized lower dentition [15], [16], with modification of the architecture of the mandible [7], [17] and bones of the masticatory apparatus [6]. Accordingly, tree-gouging is characterized by the use of the maxillary teeth to puncture and hold onto a plant branch and the mandibular incisors to scrape the branch [12], [13]. These characteristics enable gouging animals to drill holes of various shapes and sizes to reach ducts in the plant tissue and release the exudates [12], [13], [18]–[20]. Additionally, marmosets have evolved a specialized system for digesting exudates through fermentation, as their cecum and colon are disproportionately large compared to the rest of their body [14], [21]. This is an important feature because fermentation by microorganisms is essential for the extraction of energy from the complex polysaccharides contained within plant exudates [11], [22].

The use of exudates as a food has implications for the ecology and social organization of primates [1], [3], [18]. The predictability of periodic amounts of gums confers a competitive advantage to these primates, who have relatively high energy demands because of their small size [1]. The availability of exudates favors the social use of a food resource [3], with more exudativorous species inhabiting smaller areas because of the predictable and more or less constant presence of their food source. Apart from metabolism, exudativory also appears to have an impact on the development of psychological differences among species [23].

Exudates are significant sources of complex carbohydrates, proteins and certain minerals, especially calcium [1], [4], [24]–[26]. *Callithrix* species often extract exudates from trees of the genus *Anadenanthera* (Fabaceae) especially *Anadenanthera peregrina* var. *peregrina* (L.) Speg. [19], [20], [27]–[29]. Species of the genus *Anadenanthera* are widely distributed in the phytogeographical areas of the Caatinga, Cerrado (Brazilian savannah), and Atlantic Forests in the Northeast, Midwest, and Southeast regions of Brazil [30].

The exploitation of *Anadenanthera* spp. by marmosets is surprising because this plant can produce tannin, dyes, timber, medicinal, and psychoactive products [31]–[35] and has relatively hard bark. *Anadenantgera* spp. is sometimes covered with prickles, and high tannin levels [31], [34], [36], both which can repel primates [37]. However, these characteristics do not appear to dismay certain *Callithrix* species in obtaining exudate a from this plant. Such species include *C. penicillata* É Geoffroy, 1812 [18], [20], [38], *Callithrix jacchus* L., 1758 [18], [19], [28], [29] and *Callithrix flaviceps* Thomas, 1903 (Primates: Cebidae) [27], [39]. *Callithrix* species are well known for consuming exudates from plants, but their exploitation of gum produced by trees, in particular, is poorly studied. However, this information is important for understanding the relationships between the behavioral and ecological traits of marmosets and their target tree species.

The study of plant–animal interactions has a key role in ecological theories and in the understanding issues related to the conservation of biodiversity. This is the first study to describe the ecological relationships between marmosets and their target trees and this work contributes to a better understanding of the exudativory pressure exerted by these primates. Here we specifically evaluate the intensity of exploitation of exudates from *A. peregrina* var. *peregrina* by *Callithrix* spp. in forest fragments of the Atlantic Forest in the state of Minas Gerais, Brazil. We also determine the preferred foraging location on the tree and the number of holes made to obtain exudates, and link this information to dendrometric data (DBH diameter at 1.3 m height and total height) of *Anadenanthera peregrina* var. *peregrina*.

## Materials and Methods

### Study areas

The study was conducted in areas used by five marmosets groups, each with six to twelve hybrid individuals with intermediate characteristics of *C. penicillata* × *C. jacchus* and *C. penicillata* × *Callithrix geoffroyi*. The study of tree orifices gouged by hybrid marmosets is complementary to previous studies of the behavioral ecology of these animals [12], [40]. Moreover, populations of hybrid marmosets are increasing in number in the Atlantic Forest as a result of anthropogenic environmental disturbance and illegal trafficking of wild animals [41].

**Figure 1:**
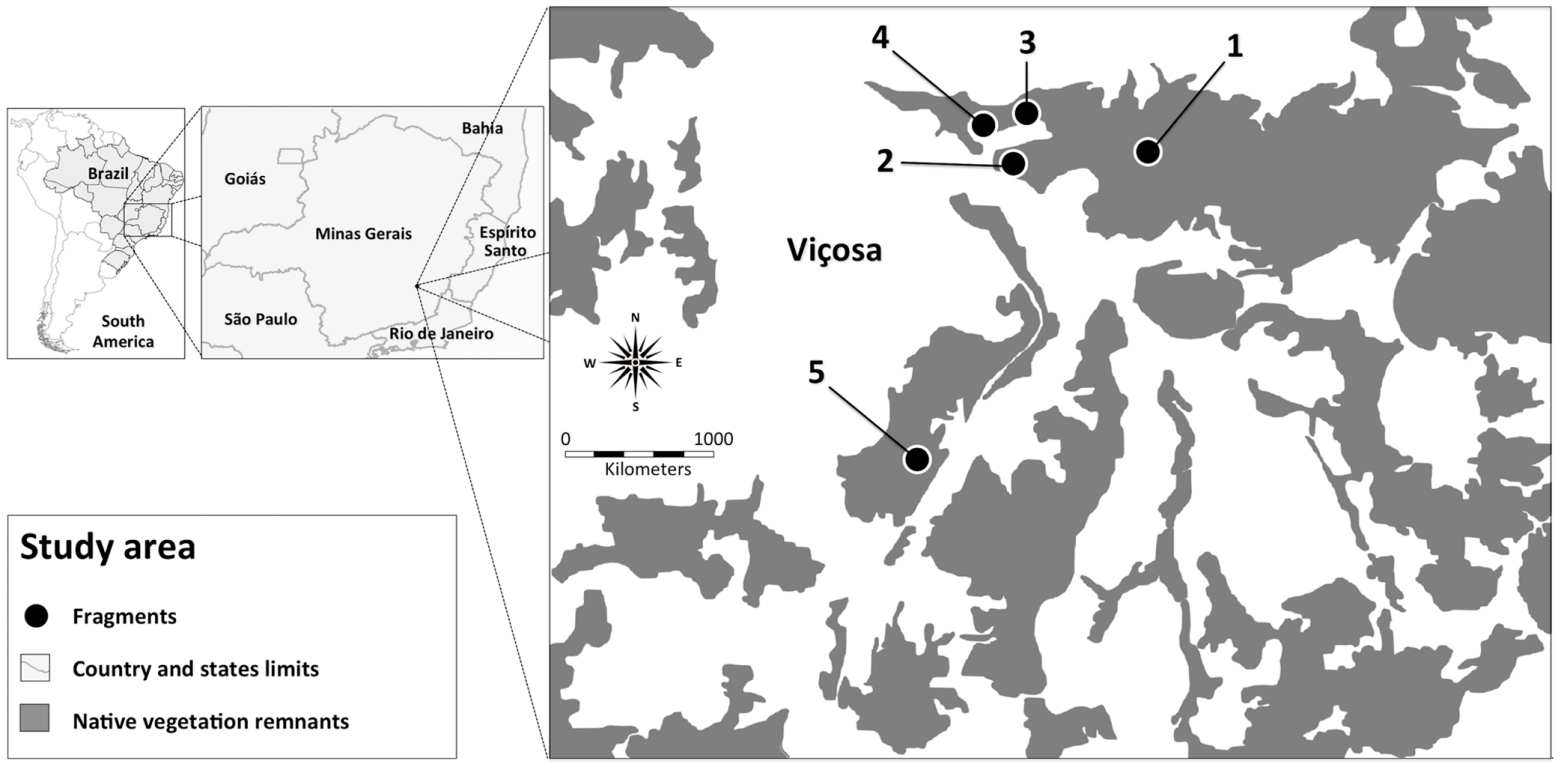
Location of the five forest fragments of the Atlantic Forest in Viçosa, Minas Gerais State, Brazil, where the study was conducted.

Our study areas referred to as Fragments 1 (20°45′34.71″S, 42°51′57.84″W); 2 (20°45′22.28″S, 42°52′23.81″W); 3 (20°45′11.16″S, 42°52′16.80″W); 4 (20°45′13.85″S, 42°52′26.90″W), and 5 (20°46′17.41″S, 42°52′37.03″W) (Fig. 1) were located on the campus of the Universidade Federal de Viçosa, Viçosa, in the state of Minas Gerais State, Brazil, at 675 to 709 m above sea level.

The region studied has montane seasonal semideciduous forest [42] and a highland tropical climate with rainy summers and cold, dry winters, which is classified as ‘Cwb’ based on the Köppen climate classification [43]. The area's average annual rainfall and temperature are 1221 mm and 19°C, respectively [44].

### Data collection and analysis

*Anadenanthera peregrina* var. *peregrina* was selected as the focal study tree because it is the only tree species exploited by the groups of *Callithrix* spp. present within the study area.

**Figure 2:**
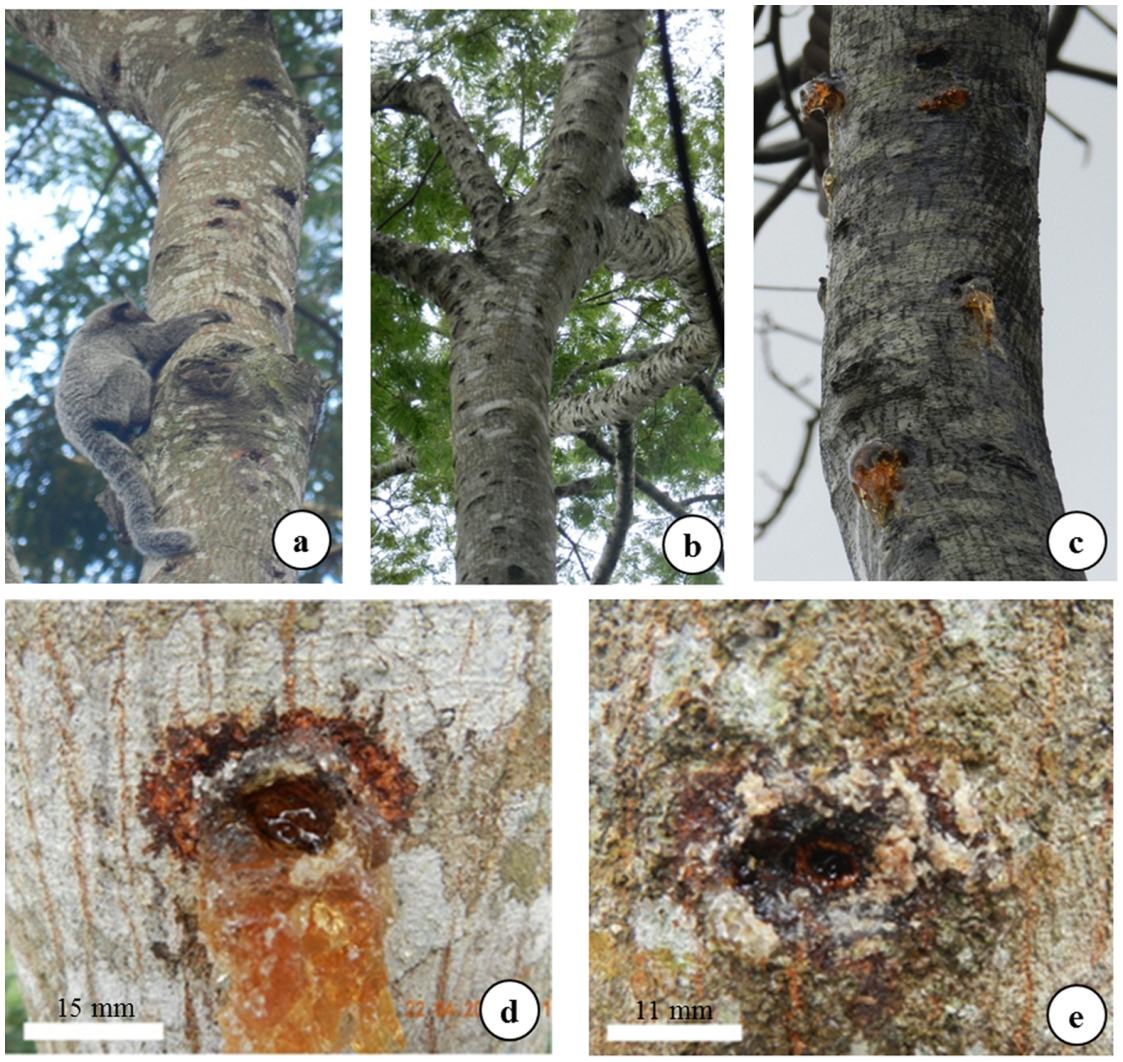
Exploration of *Anadenanthera peregrina* var. *peregrina* (Fabaceae) exudates by *Callithrix* spp. (Callitrichidae) hybrids in fragments of Atlantic Forest in Minas Gerais State, Brazil, where: (a) Hybrid *Callithrix* spp. scarifying hole, (b) detail of the canopy branches extensively explored, (c) branches with exudate released (d and e) detail of the holes with exudate.

Thirty-nine *A. peregrina* var. *peregrina* trees exploited by marmosets were sampled (Fig. 2). These trees were marked with sequentially numbered aluminum plates and the tree holes were measured and quantified with rock climbing equipment and techniques adapted for canopies [45]. This method enabled data collection from the higher and less accessible parts of the tree. The total tree height was measured with a hypsometer (Suunto PM 5, Finland) and the DBH with a diameter tape.

Plants with holes, which characterize its use by marmoset were collected for identification, except those that were difficult to access.

The fertile parts (branches with flowers and fruits) of four voucher specimens were collected in the field, herborized with floristic methodology, and sent to relevant experts for identification. This material was incorporated into the collection of the Herbarium of the Universidade Federal de Viçosa with the numbers VIC, 38241; 38240; 38239; and 38615.

**Figure 3:**
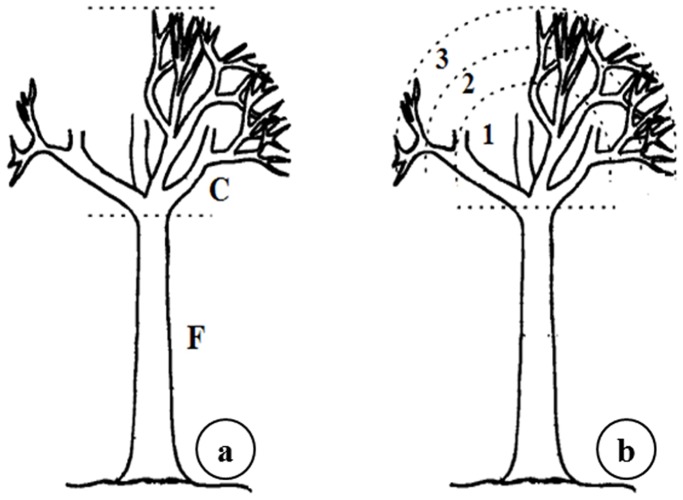
Scheme of the division of *Anadenanthera peregrina* var. *peregrina* (Fabaceae) trees in ecological zones to quantify the exploitation of exudates by *Callithrix* spp. (Callitrichidae) in fragments of Atlantic Forest in Minas Gerais State, Brazil. Division of the tree (a) in two ecological zones (F- trunk, C- canopy) and of the canopy (b) in three segments (1- lower canopy; 2- midlle canopy; 3- upper canopy).

Individuals of *A. peregrine* var. *peregrina* were divided into two ecological zones: the trunk and the canopy [46] (Fig. 3a), with the canopy subdivided into three segments: lower, middle, and upper canopy [46], [47] (Fig. 3b). All holes on the trunk were counted and measured, whereas those in the canopy were counted and measured as high up as it was safe for the researcher to go. Therefore, the percentage of holes was estimated for the canopy. The basal and distal diameter and the total length of the branches of the canopy sampled were also measured and an average diameter was obtained for the sampled branches.

Gouged tree holes were classified as inactive, characterized by presence of scar tissue and as active, without presence of scar tissue. The size of holes was measured with a digital caliper and their height (to the bark, in a vertical stroke), width (up to the bark, in a horizontal stroke), and depth (deepest portion in the hole) recorded. These parameters were obtained for active holes only, because these were assumed to be currently exploited by the marmosets. All the holes were counted, including those with scar tissue and those that were not being used by the animals. The intensity of marmoset exploitation of *A. peregrina* var. *peregrina* plant excudates was assessed by: (1) the number of active and non-active holes, and (2) height, width, and depth of the active holes.

The relation between the total number of gouged holes and the trunk diameter at 1.3 m from the soil (DBH) and tree height was analyzed by using the Pearson correlation test. The chi-square test was applied to determine whether the number of active and non-active holes differed between the three canopy segments. Analysis of variance (ANOVA) and *post hoc* Tukey test was used to assess whether the average diameter of the branches of each canopy segment and the dimensions of the active holes differed between these segments. Differences between the volumes of active holes in the trunk versus those in the canopy were evaluated with Student’s t-test. All analyses were carried out using the computer program R 3.0.1. [48].

No specific permits were required for to study *A. peregrina*var. *peregrina* and *Callithrix* spp. in Brazil. The field studies did not involve endangered or protected species.

## Results

Measurements from 39 *A. peregrina* var. *peregrina* trees recorded ranges of DBH and total height of 6.8–64.9 cm and 6.9–35.5 m, respectively. We counted a total of 8,765 gouged holes, with 970 (11%) of holes located in the trunk and 7,795 (89%) holes located in the canopy. The total number of holes per tree ranged from 8 to 2288.

Hybrid marmosets preferrentially obtained exudates from branches in the canopy of *A. peregrina* var. *peregrina*. The upper canopy showed the higher number (*x^2^* = 143.38; p<0.001) of both active and inactive holes (48%) followed by middle (30%) and lower canopy (22%).

A separate analysis of active gouge holes showed the same type of variation in the three regions of the tree canopy (Figure 3), showed that there was a significant difference in means of tree holes present in the three different portions of the canopy in the upper. (ANOVA, df = 2, F = 53.45, p<0.001). In the upper canopy, the average branch length was smaller (32.19±0.95; p<0,001) than those in the middle canopy (39.36±1.33 cm) and lower canopy (53.30±2.03 cm) respectively.

Of the 915 active holes recorded, 810 (89%) were in the canopy and 105 (11%) were in the trunk (Table 1). The lower canopy had a smaller number and dimension of holes than the upper canopy (Table 1). The marmosets exploited the upper canopy more than the lower canopy to obtain exudates (Table 2).

**Table 1:** Height, width, depth and number (Num.) of active holes scarified by hybrid marmosets in the stem and canopy of *Anadenanthera peregrina* var. *peregrina* (Fabaceae).

Parameters	Tree Zone	Num.	Average (mm) ± standard error
Height	Trunk	105	10.21±0.18
	Canopy	810	11.70±0.10
Width	Trunk	105	14.13±0.28
	Canopy	810	18.49±0.24
Depth	Trunk	105	4.98±0.26
	Canopy	810	7.05±0.09

Student’s t-test (p<0.01) (n = 915).

**Table 2:** Height, width, depth and number (Num.) of active holes scarified by hybrid marmosets in the lower, middle and upper canopy of *Anadenanthera peregrina* var. *peregrina* (Fabaceae).

Parameters	Canopy segments	Num.	Average (mm) ± standard error
Height	Lower	145	10.35±0.19
	Midlle	377	11.63±0.15
	Upper	288	12.45±0.16
Width	Lower	145	16.01±0.49
	Midlle	377	17.92±0.29
	Upper	288	20.48±0.48
Depth	Lower	145	5.73±0.17
	Midlle	377	6.93±0.14
	Upper	288	7.88±0.17

ANOVA, Tukey test, *post hoc* (p<0.01) (n = 810).

The total number of gouged holes showed weak to moderate correlation with the DBH (Pearson correlation; r^2^ = 0.530) and total height (Pearson correlation; r^2^ = 0.435 of trees). These correlations suggest a positive relationship between concentration of gouged holes and their location in the canopy and that marmoset preferably obtain exudates from canopy branches in the canopy of *A. peregrina* var. *peregrina*.

## Discussion

The feeding by marmosets on gum from holes in trees depends on various tree features. *Anadenanthera peregrina* var. *peregrina* are among the 80 species identified as sources of exudates exploited by *Callithrix* spp. [2]. The high number of scarifications on this tree species recorded in this study highlights it as a preferred source of food for marmosets; in addition, no scarifications were found on the other plant species such as *Tapirira guianensis* Aubl., *Allophylus edulis* Radlk. ex Warm.; *Astronium fraxinifolium* Schott, which are used by *C. jacchus*. However, the use of only one plant species differs from the results in the Caatinga and Cerrado, where *Callithrix jacchus* and *C. penicillata* exploited a larger number of gum trees species [20], [28], [29], [49]. The greater number and size of holes in the canopy compared with those in the trunk can be explained by the smaller branch diameter in the canopy. The positive correlation between the number of scarifications with DBH and tree height may be explained by a larger area to be exploited to obtain exudates.

The exclusive use of *A. peregrin*a var. *peregrin*a by hybrid marmosets to obtain exudates in the five forest fragments and the absence of scarification on other tree species merits further study. Our results particularly raise the question if there are particular nutrients present in *A*. *peregrina* that are lacking other plants to explain the exclusive use of just a single plant species. Further study of this question would help us understand the marmosets selection and exploitation of exudate resources. It is known that the exudates of *Anadenanthera* have high concentrations of polysaccharides and calcium [50], [51] and exudate polysaccharides are an important energy source for marmosets [52]. Calcium plays an important role to maintain the calcium/phosphorus metabolism balance of organisms [53]. Female marmosets typically give birth to twins twice a year, and calcium may be especially important during pregnancy and milk production for these animals [18], [54]. Thus, one possibility for the exclusive utilization of *Anadenanthera* by marmosets in our study may be a unique role of the tree species to fulfill the dietary and energetic needs of these primates.

The use of exclusive trees by *Callithrix* spp. to obtain exudates is uncommon given that, generally, these species use a large number of gum tree species to obtain exudates. *Phaner furcife*r Blainville, 1839 in Madagascar [55], *Nycticebus coicang* Boddaert, 1875 in West Malaysia (Manjung District, Perak State), *Cebuella pygmaea* in Northeastern Ecuador [56] and *C. pygmaea* in Iquitos, Peru [57] fed on 10, 9, 18 and 58 plant species, respectively. In the Brazilian Cerrado, *Callithrix penicillata* used 14 gum tree species [2], [20], [58], [59] and this same number was reported to have been used by *Callithrix jacchus* in the Caatinga [28], [29]. The scarce availability of resources in these ecosystems, with more extreme climatic conditions, could explain the use of different plant species [18]. However, *C. jacchus* explores mainly exudates from *Anadenathera peregrina* [28] in the Caatinga, a biome with poor gum tree diversity. Therefore, further phytosociological studies of *A. peregrina* in different habitats are necessary to explain patterns of marmoset preference for gum trees.

The marmosets evaluated in this study fed only exudates of *A. peregrina* var. *peregrina*, despite the fact that the study areas were inhabited by other plant gum trees, e.g., *Tapirina guianensis* Aubl. used for *C. jacchus* [13], [18]; *Callithrix kuhlii* Coimbra Filho, 1985 [60]; *C. penicillata* [49]; *Mico melanurus* (É. Geoffroy in Humboldt, 1812) [61], *Piptadenia gonoacantha* (Mart.) J.F. Macbr. used for *C. flaviceps* [62] and *Astronium fraxinifolium* Schott. used for *C. jacchus* [18], [63]; which show a preference of marmosets for *A. peregrina* var. *peregrina.*

The absence of scarification on some *A. peregrina* var. *peregrina* plants is similar to the pattern for other plant gums as *Vochysia pyramidalis* Mart., *Callisthene major* Mart. & Zucc. and *Tapirina guianensis* Aubl. used by *C. penicillata* [2] and on *Anacardium occidentale* L. [18], *Anadenhantera peregrina* (L.) Speg., *Astronium fraxinifolium* Schott, *Enterolobium contortisiliquum* (Vell.) Morong, and *Coccoloba* sp. by *C. jacchus* [28].

The preference for certain trees of the same species can be explained by differences between the trees, such as increased production and nutritional quality of exudates, smaller amount of secondary metabolites, such as tannins, and greater protection from predators [1], [26], [55], [58].

The preference of the animals for the canopy, as shown by the number and dimensions of the holes between the ecological zones of the tree (trunk and canopy) in *A. peregrina* found in this study, might be related to the presence of thinner branches in this region, which are preferred by marmosets to exploit this resource. This preference also might be the result of physical and mechanical factors, such as a thicker bark, and properties that facilitate scarification by these animals [6]. The quantity and nutritional quality of exudates in the canopy might also be better because of a higher metabolic rate in this part of the tree and a lower quantity of secondary compounds [31], [34]. However, in ecosystems with more extreme environmental conditions, such as, the Caatinga and Cerrado, *Callithrix* spp. (*C. jacchus* and *C. penicillata*) use both the trunk and canopy of gum trees [18], [20], [28], [49], [59], with a greater number of holes found in the trunk [28], [49]. Thus, the strategy of using such tree species by *C. jacchus* and *C. penicillata* might be affected by environmental conditions [28], [59]. Similarly, *Nycticebus* spp. *pygmaeus* Bonhote, 1907 (Primates: Lorisidae) made more use of the trunk than of the canopy for exudate feeding in a mixed deciduous forest in the Seima Protection Forest, Eastern Cambodia [5].

The increased preference of marmosets for the apical segments of the canopy, with a higher percentage of exudates and larger scarified holes, agrees with observations made between canopy segments. The increased branch use in the external parts of the canopy, where younger branches are found, reflects a preference for plant parts that are less thick, facilitating scarification. The preference of marmosets for tree segments with flatter branches horizontally and thinner bark reduces the energy used for foraging and effort in obtaining exudates. Marmoset species such as *C. penicillata* and *C. jacchus* have a bimodal pattern of exudate exploitation, with peaks in the morning and late afternoon [38], [64]. After consuming the exudates, the marmosets scarify the bark and holes in parts of the tree that have a higher metabolic rate, justifying the exploration of more exposed and thinner branches of the canopy. The benefits of acquiring the exudates must be higher than the associated costs, which will depend on the energy required to keep the marmosets in the canopy and involved in scarification. These factors both have an impact on the energy spent by marmosets in obtaining exudates, as reported for other primate species ) [65], [66].

The forest fragments used by marmosets are inside an anthropogenic matrix, surrounded by urbanized areas and/or through-ways for humans, which could have a negative impact on the animals [67]–[70]. The marmosets might have a lower predation risk in these areas, facilitating the exploration of exudates in the canopy, especially those in the outside part of the trees. Furthermore, exploration of the external canopy to acquire exudates is favored by the small size of the animal.

The positive relation between the number of scarified holes and the dendrometric data of trees (DBH and total height) agrees with results observed previously for *C. penicillata* and *C. jacchus* on *Qualea parviflora* Mart [59], *Astronium fraxinifolium*; *Enterolobium contortisiliquum,* and *Anadenanthera peregrina* [28]. This result suggests that larger trees tend to be more heavily scarified by *Callithrix*, and this choice is related to the ability of these animals to climb trees and the larger area available for scarification. However, the low correlation between the number of holes and DBH and tree height indicates that this preference is not determined by tree size, but by other factors, such as bark thickness and the chemical composition of the exudates [1], [26].

The preference of *Callithrix* sp. for *A. peregrina* var. *peregrina* exudates in the Atlantic Forest differs from the pattern for these animals in the Caatinga and Cerrado forests, where primates, in general, exploit many species of gum trees [28], [29], [49], [58]. The external canopy is further explored by these animals to obtain this resource with a positive correlation between the number of scarifications and DBH and tree height.

The high number of scarifications highlights the importance of *A. peregrina* var. *peregrina* as a source of exudates for *Callithrix* spp. These results contribute to understanding of the selective pressures exerted by marmosets on certain tree species and individuals to obtain this valuable resource.
